# Hydroxylated-Benz[a]anthracenes Induce Two Apoptosis-Related Gene Expressions in the Liver of the Nibbler Fish *Girella punctata*

**DOI:** 10.3390/toxics12120915

**Published:** 2024-12-18

**Authors:** Muhammad Ahya Rafiuddin, Hajime Matsubara, Kaito Hatano, Masato Honda, Kenji Toyota, Kouhei Kuroda, Keito Tsunoda, Yukihiro Furusawa, Yoshiaki Tabuchi, Tetsushi Hirano, Akihiro Sakatoku, Chun-Sang Hong, Ajai K. Srivastav, Thumronk Amornsakun, Nobuaki Shimizu, Mohamed I. Zanaty, Tatsuo Harumi, Kohei Yamauchi, Tamás Müller, Ning Tang, Atsuhiko Hattori, Kazuichi Hayakawa, Nobuo Suzuki

**Affiliations:** 1 Noto Center for Fisheries Science and Technology, Kanazawa University, Ossaka, Noto-cho, Ishikawa 927-0552, Japan; 2 Noto Marine Laboratory, Institute of Nature and Environmental Technology, Kanazawa University, Ogi, Noto-cho, Ishikawa 927-0553, Japan; 3 Botanical Garden, Institute of Nature and Environmental Technology, Kanazawa University, Kanazawa, Ishikawa 920-1192, Japan; 4 Department of Bioresource Science, Graduate School of Integrated Sciences for Life, Hiroshima University, 1-4-4 Kagamiyama, Higashihiroshima, Hiroshima 739-8528, Japan; 5 Department of Pharmaceutical Engineering, Faculty of Engineering, Toyama Prefectural University, Imizu, Toyama 939-0398, Japan; 6 Division of Molecular Genetics Research, Life Science Research Center, University of Toyama, Sugitani, Toyama 930-0194, Japan; 7 School of Science, Academic Assembly, University of Toyama, Gofuku, Toyama 930-8555, Japan; 8 Graduate School of Particulate Matter Management, Korea University, Seongbuk-gu, Seoul 02841, Republic of Korea; 9 Department of Zoology, D.D.U. Gorakhpur University, Gorakhpur 273-009, India; 10 Fisheries Technology Program, Faculty of Science and Technology, Prince of Songkla University, Pattani 94000, Thailand; 11 Biotechnology and Life Sciences Department, Faculty of Postgraduate Studies for Advanced Sciences, Beni-Suef University, Beni-Suef 62511, Egypt; 12 Department of Anatomy, Asahikawa Medical College, Asahikawa, Hokkaido 078-8510, Japan; 13 Department of Freshwater Fish Ecology, Institute of Aquaculture and Environmental Safety, Szent István Campus, Hungarian University of Agriculture and Life Sciences, 2100 Godollo, Hungary; 14 Institute of Nature and Environmental Technology, Kanazawa University, Kakuma-machi, Ishikawa 920-1192, Japan; 15 Department of Sport and Wellness, College of Sport and Wellness, Rikkyo University, Niiza, Saitama 352-8558, Japan; 16 Low Level Radioactivity Laboratory, Institute of Nature and Environmental Technology, Kanazawa University, Nomi, Ishikawa 923-1224, Japan

**Keywords:** benz[a]anthracene, hydroxylated-benz[a]anthracene, apoptosis-related factors, biomarkers, *Girella punctata*

## Abstract

Polycyclic aromatic hydrocarbons (PAHs) are known to have toxic effects on fish. In this study, we examined the effects of benz[a]anthracene (BaA), a type of PAH, on fish liver metabolism. Nibbler fish (*Girella punctata*) were intraperitoneally injected with BaA (10 ng/g body weight) four times over a 10-day period. BaA significantly decreased known bone metabolism-related plasma factors such as calcium and inorganic phosphorus. Moreover, significant reductions were observed in the plasma levels of known liver metabolism-related factors, including ferrous ions, total bile acids, total bilirubin, free bilirubin, aspartate aminotransferase, and alkaline phosphatase. Interestingly, mono-hydroxylated metabolites of BaA, such as 3 hydroxylbenz[a]anthracene (3-OHBaA), were detected in the bile of BaA-injected nibbler fish. This hydroxylated form of BaA was found in its free form, rather than conjugated with glucuronic acid or sulfuric acid. Due to the lack of whole-genome sequence data for the nibbler fish, two nibbler fish-specific apoptosis-related factors (TNF receptor superfamily member 1A: *tnfrsf1a* and TNF superfamily member 10: *tnfsf10*) were isolated by De novo RNA sequencing. In a liver tissue culture, 3-OHBaA (10^−6^ M) significantly upregulated the expression of *tnfrsf1a* and *tnfsf10* in the liver. These results provide the first evidence that 3-OHBaA metabolites exhibit toxic effects on the liver in teleost.

## 1. Introduction

Polycyclic aromatic hydrocarbons (PAHs) are ubiquitous environmental pollutants derived from petroleum, produced through the incomplete combustion of fossil fuels, wood, and other organic materials [1]. Major oil spills, such as those from the Deepwater Horizon and sunken oil tankers (Exxon Valdez in Alaska, USA, and Nakhodka in Fukui prefecture, Japan), have caused significant ocean pollution, impacting marine organisms [2,3,4,5,6]. For over 14 years following the Exxon Valdez spill, the toxicity of PAHs from the oil persisted in marine animals [7]. The Deepwater Horizon disaster released over 636 million liters of crude oil into the Gulf of Mexico, affecting fish embryos, with PAHs in the oil causing acute and delayed mortality in fish due to cardiotoxicity [6,8].

Benz[a]anthracene (BaA), a type of PAH, has been detected in sediments from specific aquatic environments worldwide, with mass ratio ranging from 20 to 1450 ng/g of dry weight, including the Pearl River (China), Seine River (France), Mersey River (UK), Lenga River (Chile), Black Sea (Ukraine), Dar es Salaam Coast (Tanzania), Lake Superior (Canada), and Pacific Northwest lakes (USA) [9]. BaA has also been detected in the muscle tissue of several fish species [10,11,12], such as European catfish (*Silurus glanis*) from Italy’s Po River, with BaA levels ranging from 0.05 to 0.3 ng/g of fresh weight [10], and in the liver of juvenile coho salmon (*Oncorhynchus kisutch*) from Pacific Northwest lakes, with BaA levels of 2 to 18 ng/g fresh weight [13]. These findings suggest that BaA in the environment is readily accumulated by fish and accumulates in their muscles and liver.

Previously, we injected BaA intraperitoneally into marine nibbler fish (*Girella punctata*) over a 10-day period [14]. The results showed that high-dose BaA exposure significantly induced hypocalcemia and hypophosphatemia, along with a decrease in osteoblast (bone-forming cells) and osteoclast (bone-resorbing cells) biomarkers. Beyond disrupting bone metabolism, BaA exposure also led to a significant reduction in liver metabolic enzyme activities, including alkaline phosphatase (ALP) and aspartate aminotransferase (AST), indicating possible impacts on liver function in nibbler fish.

Previous studies have shown that 3-hydroxy BaA (3-OHBaA) can affect bone metabolism in fish [15]. Moreover, Suzuki et al. [16] demonstrated that 3-hydroxylated benzo[c]phenanthrene (3-OHBcP), another type of PAH, affects osteoblast apoptosis in fish.

Based on these findings, this study focused on BaA’s effects on liver apoptosis and investigated whether BaA is hydroxylated and how it affects the expression of liver apoptosis-related genes in nibbler fish.

## 2. Materials and Methods

### 2.1. Animals

Nibbler fish were captured from Tsukumo Bay on the Noto Peninsula (Ishikawa Prefecture, Japan) from April 2020 to August 2024. The present study was approved by the Guide for the Care and Use of Laboratory Animals at Kanazawa University (AP24-010, approved on 11 April 2024). Before 2024, as we only reared juvenile fish, a separate ethics certificate was not required.

### 2.2. Plasma and Bile Samples from Intraperitoneal Injection with BaA in Nibbler Fish

Immature nibbler fish (n = 23; mean weight, 74.8 ± 5.2 g) were divided into control (n = 11) and experimental (n = 12) groups. Fish were anesthetized with 2-phenoxyethanol (0.04%, FUJIFILM Wako Pure Chemical Co., Ltd., Osaka, Japan), and BaA(FUJIFILM Wako Pure Chemical Co., Ltd., Osaka, Japan) was injected intraperitoneally at a dose of 10 ng/g body weight, which was determined from the concentration in environmental water [9], the concentration in fish muscle [10,11,12,13], and the prior study [14]. The injections were administered four times, on days 1, 3, 6, and 9. BaA was initially dissolved in ethanol, with 0.9% NaCl added to reach a final ethanol concentration of 0.1%. Control fish received saline (0.9% NaCl with 0.1% ethanol) in the same manner as the experimental group. Fish were maintained at 26 °C for 10 days and fed commercial marine fish food (Marubeni Nisshin feed Co., Ltd., Tokyo, Japan) daily in the morning. Just 10 days after the beginning of the experiment, the fish were anesthetized again. Blood samples were collected from the caudal blood vessel of the anesthetized fish using a heparinized syringe. Then, bile was collected from the gallbladder with a syringe. The collected blood was placed into 1.5 mL tubes, which were then centrifuged at 5200× *g* for 3 min. Plasma and bile were immediately frozen and stored at −80 °C until further analysis.

### 2.3. Measurement of Plasma Calcium (Ca) and Inorganic Phosphorus (iP) Levels in Nibbler Fish

Plasma Ca levels (mg/dL) were determined using a Ca II assay kit (Shino-Test Corporation, Tokyo, Japan). The plasma iP levels (mg/dL) were analyzed with the IP-II assay kit provided by Kyowa MEDEX Co., Ltd. (Tokyo, Japan).

### 2.4. Measurement of Plasma Total Bile Acids, Total Bilirubin, Free Bilirubin, ALP, AST, and Minerals in Nibbler Fish

Plasma samples were sent to a commercial analysis laboratory (Oriental Yeast Co., Ltd., Tokyo, Japan). The plasma levels of total bile acids, total bilirubin, free bilirubin, and Fe^2+^, as well as the activities of ALP and AST, were measured using specific kits from FUJIFILM Wako Pure Chemical Co. Ltd. The plasma levels of Na^+^, K^+^, and Cl^−^ were quantified using an ion electrode method with a Hitachi 7180 automatic analyzer (Hitachi High Technologies Corporation, Tokyo, Japan).

### 2.5. Detection of 3-OHBaA in Nibbler Fish

Bile, enzymatically pretreated according to Suzuki et al. [17], was measured as follows: As outlined by Honda et al. [18], the bile samples were pretreated with the QuEChERS (Quick, Easy, Cheap, Effective, Rugged, and Safe) method using the AOAC (Association of Official Analytical Chemists) extraction packet method and 15 mL dispersive Solid-Phase Extraction for fatty samples (Agilent Technologies, Santa Clara, CA, USA). Following this, the 3-OHBaA metabolites were analyzed using an LC-MS/MS system (API-4500; AB Sciex, Milford, MA, USA) with a Kinetex F5 column (250 × 2.1 mm, 5 µm; Phenomenex, Torrance, CA, USA).

### 2.6. Toxic Effect of 3-OHBaAs on Cultured Liver Tissue of Nibbler Fish

Following anesthesia, the livers of non-mature nibbler fish (n = 5; mean weight, 564.0 ± 55.2 g) were carefully collected. The liver tissue pieces were then divided into four groups: two treated with 3-OHBaA (10^−6^ M) and two control groups. The liver tissue pieces were placed in Leibovitz’s L-15 Medium (Thermo Fisher Scientific, Waltham, MA, USA) containing 1% penicillin–streptomycin mixture (Thermo Fisher Scientific) and cultured at 15 °C for 6 h. After incubation, the liver samples were preserved in RNALater^TM^ (Sigma-Aldrich Inc., St. Louis, MO, USA) and stored at −80 °C.

### 2.7. De Novo RNA Sequencing (RNA-Seq) Analysis and Selection of Apoptosis-Related Genes

Since there are no available whole-genome sequence data for nibbler fish, we performed de novo RNA seq to identify apoptosis-related genes. Eleven immature nibbler fish (mean weight, 70.7 ± 8.2 g) were used for RNA-seq analysis to construct gene models related to apoptosis. The livers of these fish were collected under anesthesia. Liver total RNA extraction was performed using a NucleoSpin RNA II kit (Takara Bio Inc., Otsu, Shiga, Japan). Following the method of Hatano et al. [19], cDNA libraries from liver total RNA were constructed and the library was sequenced using an Illumina NovaSeq 6000 platform (Illumina, San Diego, CA, USA) with a 150 bp paired-end module. The quality of the resulting sequences was assessed using the FastQC program (version 0.11.2, available online at: http://www.bioinformatics.babraham.ac.uk/projects/fastqc (accessed on 10 September 2024). All reads were assembled using the RNA-seq de novo assembler Trinity (version 2.15.1) in paired-end mode [20]. The reference transcriptome was aligned to the NCBI protein database NR (non-redundant) using BLASTX (E-value threshold = 10^−3^) with the AC-DIAMOND package (version 2.0.15) [21]. The data were deposited in the DDBJ8 Sequence Read Archive under accession number DRR595512. The obtained data were analyzed using GeneSpring (Agilent Technologies, Inc., Santa Clara, CA, USA) to identify significant genes, and Ingenuity Pathway Analysis (IPA; Thermo Fisher Scientific, Inc., Waltham, MA, USA) to examine gene ontology, including biological processes, cellular components, molecular functions, and gene networks. Based on the obtained results, we in silico cloned tumor necrosis factor (TNF) receptor superfamily member 1A [22,23] *tnfrsf1a* and TNF superfamily member 10 [24,25,26] *tnfsf10*, two important key genes implicated in hepatic apoptosis [27]. Forward and reverse primers were designed to confirm the partial cDNA sequences encoding *tnfrsf1a* and *tnfsf10* from the nibbler fish reference transcriptome DRR595512 sequence.

*tnfrsf1a*

Forward 5′-CACTTGAGACACTCATATGTTAC-3′

Reverse 5′-AGTGGTTGTCCAGGTGTC-3′

*tnfsf10*

Forward 5′-CAGTGATGACGAACTGTTCG-3′

Reverse 5′-CCCAGACCTACTTCAGACACA-3′

cDNA synthesis was then performed using the PrimeScript^TM^ II 1st Strand cDNA Synthesis Kit (Takara Bio Inc.) with the above liver total RNA extracted. The cDNA was amplified by PCR using 30 cycles at 94 °C for 10 s, 57.5 °C for 20 s, and 68 °C for 20 s on an automated thermal cycler (Mastercycler^®^ nexus X2, Eppendorf, Hamburg, Germany). PCR products were sequenced with an Applied Biosystems 3730xl DNA analyzer (Thermo Fisher Scientific). The resulting cDNA sequences were submitted to GenBank under the accession numbers LC849219 (*tnfrsf1a*) and LC849220 (*tnfsf10*).

### 2.8. qPCR for Two Apoptosis-Related Genes in 3-OHBaA-Exposed Cultured Liver

Specific qPCR primers were designed for *tnfrsf1a* and *tnfsf10* based on their respective partial nucleotide sequences using Primer3 software (version 2.6.1) [28].

*tnfrsf1a*

Forward 5′-CAGTTGCTTCTCTGACCTTC-3′

Reverse 5′-AACCTTCAGAGAAGAAGTGTCC-3′

*tnfsf10*

Forward 5′-TGTTCCTCCCAGCTCAG-3′

Reverse 5′-TGTTCCCGCTCAGCAG-3

*ef-1a* (AB874605) [14]

Forward 5′-GTATGGTCGTCACCTTTGCTC-3′

Reverse 5′-GTGGGTCGTTCTTGCTGTC-3′

Total RNA extraction and cDNA synthesis were performed as described above. PCR amplification was performed at an annealing temperature of 60 °C using the LightCycler^®^ 96 System (Roche Diagnostics K.K., Tokyo, Japan) according to Sekimoto et al.‘s protocol [29]. The expression levels of each mRNA were normalized to the *ef-1a* mRNA level.

### 2.9. Statistical Analysis

All results are expressed as the mean ± SE. The statistical significance of the differences between the control and experimental groups was assessed using Student’s *t*-test or a paired *t*-test. In all cases, significance was set at *p* < 0.05.

## 3. Results

### 3.1. BaA Significantly Reduces Plasma Levels of Ca, iP, and Fe^2+^ in Nibbler Fish

**Figure 1:**
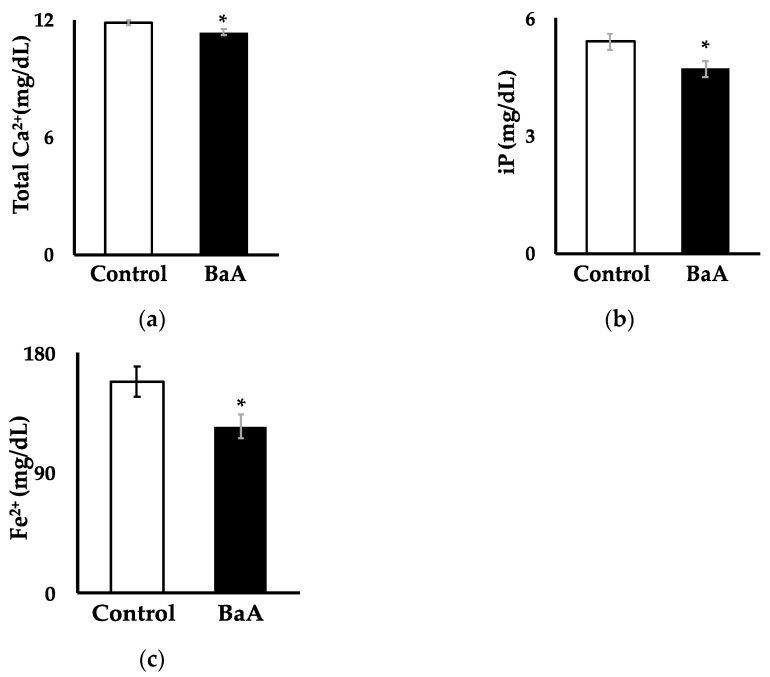
Effects of BaA on plasma Ca^2+^ (**a**), iP (**b**), and Fe^2+^ (**c**) levels in BaA-treated nibbler fish. Plasma Ca^2+^, iP and Fe^2+^ in the saline-treated control group (n = 11) are shown in white columns, and those in the BaA-treated experimental group (n = 12) are shown in black columns. Statistically significant differences, denoted by asterisks, were observed at *p* < 0.05 and were found when compared to the control group.

Figure 1a shows that the intraperitoneal injection of BaA into nibbler fish significantly reduced plasma Ca^2+^ levels (11.36 ± 0.16 mg/dL) compared to the control group (11.87 ± 0.12 mg/dL). Figure 1b indicates that BaA-treated nibbler fish experienced a significant decrease in plasma iP concentration (4.71 ± 0.20 mg/dL) relative to the control group (5.40 ± 0.19 mg/dL). Figure 1c reveals a significant reduction in plasma Fe^2+^ levels in BaA-treated nibbler fish (125.00 ± 8.99 mg/dL) compared to controls (158.55 ± 11.62 mg/dL). Conversely, no significant differences were observed between control and BaA-treated fish in plasma levels of Na⁺ (177.45 ± 0.81 mEq/L vs. 177.83 ± 1.02 mEq/L), K^+^ (3.26 ± 0.16 mEq/L vs. 3.37 ± 0.06 mEq/L), or Cl^−^ (140.09 ± 1.99 mEq/L vs. 149.17 ± 2.01 mEq/L). These results suggest that BaA may induce hypocalcemia, hypophosphatemia, and iron deficiency anemia, among other conditions. Additionally, the findings imply that BaA does not affect electrolyte disorders such as hyponatremia, hypernatremia, hypokalemia, hyperkalemia, hypochloremia, or hyperchloremia, indicating a potential disruption in liver function in this species.

### 3.2. Intraperitoneal Injection of BaA in Nibbler Fish Significantly Reduces Plasma, Total Bile Acid, Total Bilirubin, and Free Bilirubin Levels

**Figure 2:**
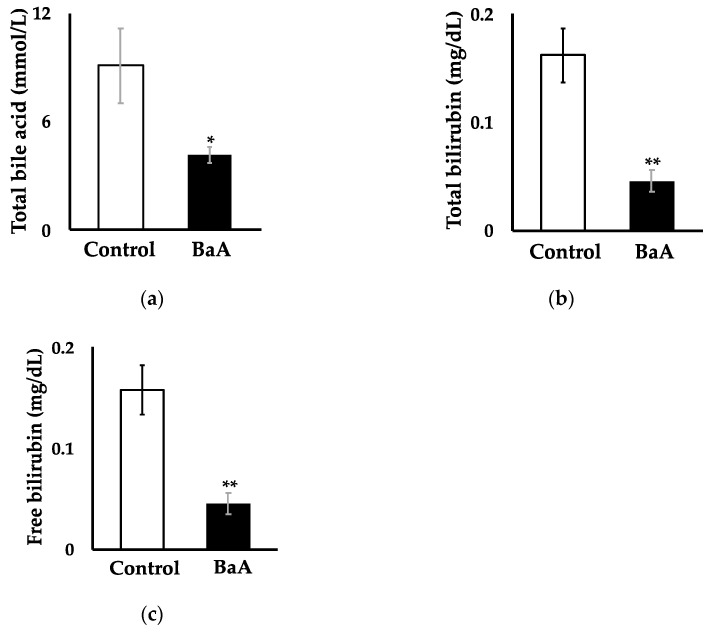
Effects of BaA on plasma total bile acid (**a**), total bilirubin (**b**), and free bilirubin (**c**) levels in the BaA-treated nibbler fish. Plasma total bile acid, total bilirubin, and free bilirubin in the saline-treated control group (n = 11) are shown in white columns, and those in the BaA-treated experimental group (n = 12) are shown in black columns. Statistically significant differences, indicated by asterisks (* *p* < 0.05, ** *p* < 0.01), were found when compared to the control group.

The intraperitoneal administration of BaA to nibbler fish significantly reduced plasma concentrations of total bile acid (4.17 ± 0.46 mmol/L; Figure 2a), total bilirubin (0.05 ± 0.01 mg/dL; Figure 2b), and free bilirubin (0.05 ± 0.01 mg/dL; Figure 2c) compared to the control group (9.09 ± 2.04 mmol/L, 0.16 ± 0.03 mg/dL, and 0.16 ± 0.02 mg/dL, respectively).

### 3.3. Intraperitoneal Injection of BaA in Nibbler Fish Significantly Reduces Plasma Levels of AST and ALP

**Figure 3:**
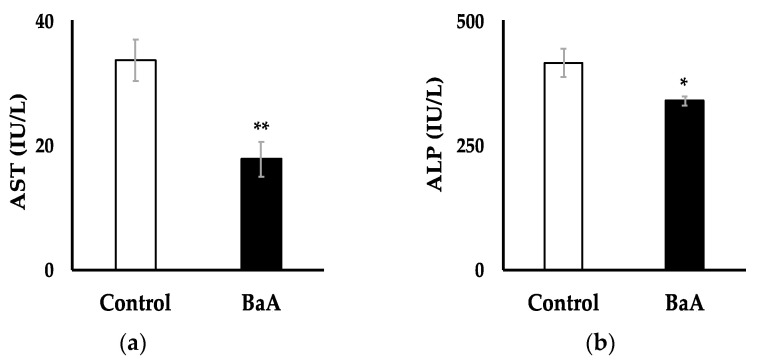
Effects of BaA on plasma AST (**a**) and ALP (**b**) activities in BaA-treated nibbler fish. Plasma activities of AST and ALP in the saline-treated control group (n = 11) are shown in white columns, and those in the BaA-treated experimental group (n = 12) are shown in black columns. Statistically significant differences, indicated by asterisks (* *p* < 0.05, ** *p* < 0.01), were observed when compared to the control group.

The plasma levels of AST and ALP, biomarkers of liver function and injury, were significantly lower in BaA-treated nibbler fish (17.75 ± 2.79 IU/L and 339.17 ± 9.66 IU/L, respectively) compared to those in the control group (33.73 ± 3.32 IU/L and 415.45 ± 29.64 IU/L, respectively) (Figure 3). The decrease in AST was accompanied by a parallel decrease in ALT, which raises concerns about potential liver failure.

### 3.4. Detection of 3-OHBaA in the Bile of BaA-Treated Nibbler Fish

**Figure 4:**
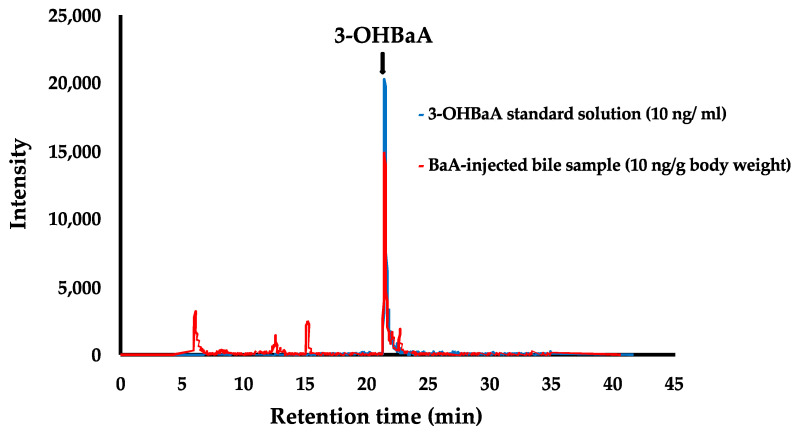
Detection of 3-OHBaA in the bile of BaA-treated nibbler fish. 3-OHBaA in the bile was not found in controls (n = 11) and was only identified in BaA-treated fish (red: n = 12). Standard 3-OHBaA solution shown by blue line.

3-OHBaA was detected in the bile of BaA-treated nibbler fish, as shown in Figure 4. The 3-OHBaA was found to be in its free form, rather than conjugated to glucuronic acid or sulfuric acid. Specifically, 3-OHBaA was identified in the bile of BaA-treated fish, while no 3-OHBaA was detected in the bile of control fish.

### 3.5. Expression of Two Apoptosis-Related Genes in 3-OHBaA-Exposed Cultured Liver

**Figure 5:**
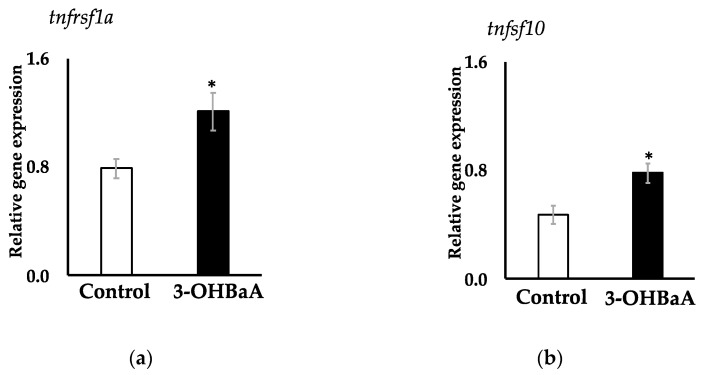
Effects of 3-OHBaA on two apoptosis-related gene expressions in the cultured liver of nibbler fish. The expression of *tnfrsf1a* (**a**) and t *tnfsf10* (**b**) in the liver is shown in white columns for the control group (n = 5) and black columns for the experimental group (n = 5). Statistically significant differences, indicated by asterisks, were observed at *p* < 0.05 compared to the control group.

De novo RNA-seq analysis generated a reference transcriptome for the nibbler fish (DRR595512), from which two apoptosis-related genes, *tnfrsf1a* and *tnfsf10*, were identified. Partial PCR products encoding *tnfrsf1a* and *tnfsf10* from nibbler fish exhibited complete sequence identity with the nucleotide (amino acid) sequences in the nibbler fish reference transcriptome DRR595512. Exposure to 3-OHBaA (10^−6^ M) significantly increased the expression of apoptosis-related genes *tnfrsf1a* and *tnfsf10* in cultured liver cells (Figure 5). Interestingly, these increases in *tnfrsf1a* and *tnfsf10* transcript abundance were accompanied by decreases in plasma levels of Ca^2+^, iP, Fe^2+^, total bile acids, total bilirubin, free bilirubin, AST, and ALP.

## 4. Discussion

BaA is a PAH commonly detected in sediments from rivers, coastal waters, and other aquatic environments worldwide. Additionally, hydroxylated PAHs are known to be metabolized from PAHs by the P4501A1 cytochrome [30] and have been detected in various organisms, including fish [2,31,32,33,34,35,36,37]. Suzuki et al. [16] found that 3-OHBcP exhibits marked toxicity by affecting bone metabolism and inducing osteoblast apoptosis in fish. In this study, we administered BaA to the marine nibbler fish (*Girella punctata*) and identified 3-OHBaA in its bile. Moreover, we observed an upregulation of apoptosis-related gene expression in the liver, suggesting that 3-OHBaA induces hepatic toxicity. These findings indicate that 3-OHBaA exerts toxic effects on the liver. Yang et al. [38] reported similar results in male Sprague Dawley rats, investigating the concentration of 3-OHBaA in urine and its hepatic toxicity. However, to our knowledge, no studies have directly examined the effects of hydroxylated PAHs on the liver in teleosts, making this study the first to investigate the direct toxic effects of BaA hydroxide on the teleost liver.

Hydroxylated BaA, specifically 3-OHBaA and 4-OHBaA, has been shown to inhibit the activity of both osteoclasts and osteoblasts in cultured scales of goldfish (*Carassius auratus*), a freshwater species, and wrasse (*Pseudolabrus japonicus*), a saltwater species [15]. Furthermore, Suzuki et al. [17] demonstrated that 4-OHBaA, a metabolite of BaA, inhibited osteoclastic (tartrate-resistant acid phosphatase) and osteoblastic (ALP) activities in goldfish scales, with 4-OHBaA detectable in the bile of goldfish following BaA administration. Additionally, BaA injection in goldfish induced hypocalcemia, hypophosphatemia, and reduced bone metabolism. These findings are similar to those in the current study, where BaA injection into nibbler fish also led to hypocalcemia, hypophosphatemia, and reduced liver metabolism. The detection of 3-OHBaA in the bile of BaA-treated nibbler fish suggests that 3-OHBaA is the primary metabolite and the main toxicant in this species. Hydroxylated PAHs have been implicated in reproductive toxicity [39], neurotoxicity [40], inflammatory responses [41], and cell damage and death pathways, including apoptosis [16].

Human hepatocyte apoptosis involves both extrinsic and intrinsic pathways, which are activated depending on various cellular conditions. The extrinsic pathway is triggered by death receptors, such as TNF receptor 1 (*tnfrsf1a,* etc.), located on the cell membrane [27]. When death ligands, like TNF (*tnfsf10,* etc.), bind to these receptors, this causes receptor trimerization, setting off a signaling cascade that activates caspase-8 and forms the death-inducing signaling complex [27]. On the other hand, the intrinsic pathway is activated by mitochondrial damage, which leads to the release of cytochrome c into the cytoplasm. This in turn activates caspase-9, which then activates effector caspases, furthering the apoptotic process [27]. Although the genomic information for nibbler fish is not yet available, the present de novo sequencing enabled the straightforward isolation of two important apoptosis-related factors, *tnfrsf1a* [22,23,27] and *tnfsf10* [24,25,26,27]. This de novo RNA-seq analysis generated a reference transcriptome for the nibbler fish (DRR595512), which is expected to contribute to various fields of future research, including toxicology. The present study demonstrated that treatment with 3-OHBaA significantly increased the expression levels of these two apoptosis-related genes in the liver, both of which play crucial roles in apoptosis regulation via above caspase activation pathways. These findings suggest that 3-OHBaA likely induces cell death in liver cells of nibbler fish, potentially contributing to apoptotic processes through these molecular mechanisms.

This study indicates that 3-OHBaA may play a role in inducing apoptosis in the liver of fish. These findings highlight the importance of future research to assess the presence and concentration of 3-OHBaA in aquatic ecosystems and fish species. BaA is estimated to be present in environmental waters at concentrations ranging from 0 to 10^−8^ M [42]. BaA has also been detected in muscle tissues of various fish species [10,11,12]. For example, BaA mass ratio in the muscle of the freshwater fish European catfish (*Silurus glanis*) range from 0.05 to 0.3 ng/g (fresh weight) [10]., while the BaA mass ratio in the liver of juvenile coho salmon (*Oncorhynchus kisutch*) ranges from 2 to 18 ng/g (fresh weight) [13]. In teleost fish, PAHs have been reported to cause reproductive toxicity [43], immunotoxicity [44], and developmental toxicity, leading to bone deformities [45,46]. Furthermore, the hydroxylated form of BcP is 1900 times more lethal than BcP itself in fish larvae [16]. These findings suggest that 3-OHBaA may also induce apoptosis in fish at low concentrations. Apoptosis is essential for maintaining normal cellular function; however, its dysregulation can lead to various harmful effects and diseases. This study demonstrated that short-term exposure to the maximum predicted environmental concentration of 3-OHBaA in nibbler fish significantly reduced liver biomarkers and upregulated the expression of two apoptosis-related genes associated with the extrinsic pathway. Prolonged exposure to 3-OHBaA may exacerbate liver cell damage, underscoring the necessity for further research to elucidate these potential effects.

In the present study, we observed that hydroxylated PAHs induce the expression of two apoptosis-related factors in the liver of marine fish. However, our findings provide only preliminary evidence regarding the involvement of hydroxylated PAHs in apoptosis within the liver. Therefore, we anticipate that a more comprehensive analysis, including the expression of additional apoptosis-related factors and histological examination of hepatocytes, will help clarify the mechanisms by which hydroxylated PAHs are linked to apoptosis in the liver.

## 5. Conclusions

In conclusion, 3-OHBaA, a metabolite of BaA, was identified in the bile of the nibbler fish (*Girella punctata*) and was found to induce significant hepatotoxic effects. Livers exposed to 3-OHBaA exhibited an upregulated expression of two apoptosis-related genes compared to controls. These findings suggest that the first documented evidence of 3-OHBaA exhibiting biological toxicity in marine fish, indicating a potential risk of liver toxicity in other vertebrates. In this study, we propose that 3-OHBaA may adversely affect the extrinsic apoptotic pathway in the fish liver. However, further investigation is required to examine its impact on the intrinsic apoptotic pathway and other related factors.

## Data Availability

The de novo RNA Sequence data discussed in this publication have been deposited in the DDBJ Sequence Read Archive, accession number DRR595512. The cDNA sequences obtained have been deposited in GenBank with the accession numbers LC849219 (*tnfrsf1a*) and LC849220 (*tnfsf10*).
